# The Voronoi theory of the normal liver lobular architecture and its applicability in hepatic zonation

**DOI:** 10.1038/s41598-021-88699-2

**Published:** 2021-04-29

**Authors:** C. Lau, B. Kalantari, K. P. Batts, L. D. Ferrell, S. L. Nyberg, R. P. Graham, Roger K. Moreira

**Affiliations:** 1 Department of Computer Science, Rutgers University, Brunswick, NJ USA; 2 Allina Health, Minneapolis, MN USA; 3 Department of Pathology, University of California, San Francisco, CA USA; 4 Division of Transplantation Surgery, Department of Surgery, Mayo Clinic, Rochester, MN USA; 5 Department of Pathology and Laboratory Medicine, Mayo Clinic, Rochester, MN USA

**Keywords:** Anatomy, Gastroenterology, Mathematics and computing

## Abstract

The precise characterization of the lobular architecture of the liver has been subject of investigation since the earliest historical publications, but an accurate model to describe the hepatic lobular microanatomy is yet to be proposed. Our aim was to evaluate whether Voronoi diagrams can be used to describe the classic liver lobular architecture. We examined the histology of normal porcine and human livers and analyzed the geometric relationships of various microanatomic structures utilizing digital tools. The Voronoi diagram model described the organization of the hepatic classic lobules with overall accuracy nearly 90% based on known histologic landmarks. We have also designed a Voronoi-based algorithm of hepatic zonation, which also showed an overall zonal accuracy of nearly 90%. Therefore, we have presented evidence that Voronoi diagrams represent the basis of the two-dimensional organization of the normal liver and that this concept may have wide applicability in liver pathology and research.

## Introduction

**Figure 1:**
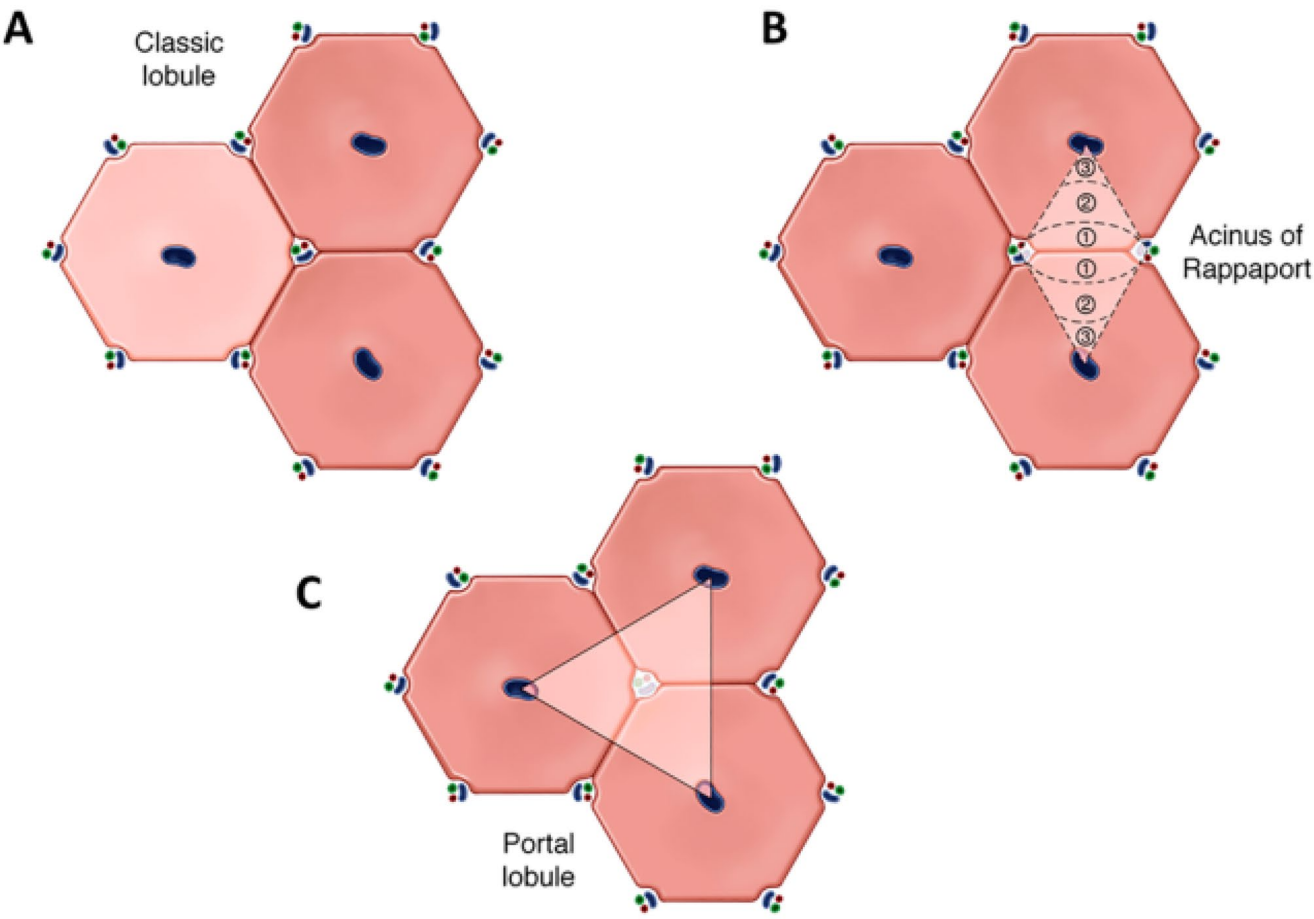
Representation of some of the different microanatomic and functional models of the liver. (**A**) Classic “Kiernan” lobule model; (**B**) acinus model of Rappaport; and C, portal lobule. Created by Mayo Clinic Medical Illustration.

The microanatomy of the liver was first described by J.J. Wepfer examining pig livers in 1665 and shortly thereafter by Malpighi in his celebrated work “*De Viscerum Structura Exercitatio Anatomica*” (cited by Kiernan^1^), in which several species were studied. Different models of the hepatic microarchitecture and their corresponding anatomic and functional units were subsequently proposed (Fig. 1). In 1833, Kiernan^1^ described and illustrated the microscopic anatomy of the liver lobules. This model would later be known as “classic” or “Kiernan” lobule, whereby the basic histologically-defined units of the liver are depicted as polygonal-shaped structures containing a central vein in the middle and portal tracts at the vertices. The boundaries of the classic liver lobules are easily recognized in some animal species (most notably in pigs—as they are often used in scientific studies—but also in camels, raccoons, and polar bears) due to the presence of well-defined fibrous septa delineating the periphery of individual normal lobules. The “hepatic acinus” model was put forth by Rappaport and colleagues^2^ in 1954, according to which the liver is subdivided in units based on terminal portal circulation—with zones 1 being closest to portal tracts, zones 3 closest to central veins, and zones 2 located between zones 1 and 3. These proposed regions, although not defined by histologic landmarks in any species, have been widely adopted in hepatology and hepatopathology due to their broad correlation with zonal patterns of expression of different cellular products, cell metabolism, as well as with zonal susceptibility to various disease processes. The Matsumoto’s “primary lobule” model^3,4^, which is based on the angio-architecture of the portal venous tree and proposes the subdivision of the classic lobules (termed “secondary lobule” in this model) into 6–8 primary lobules, has also received increasing attention and acceptance in recent decades. Other unit models have been described, including the “portal lobule”^5^ (in which the portal tracts represent the center of the lobule), the “single-sinusoid” model by Bloch^6^ and McCuskey^7^ and the cholehepaton model by Ekataksin and Wake^8^, but have not been as widely adopted as the other aforementioned models.

Recently, our group has fortuitously observed a striking similarity between the pattern of geometric partitioning of the universe illustrated by early astronomers in the seventeenth century (René Descartes, in *Principia philosophiae*^9^, supplement figure S1) and the classic lobular architecture of the liver. The mathematical basis for this method would later be described in detail by the Russian mathematician Georgy F. Voronoy (1868–1908) and referred to thereafter as Voronoi diagrams—a principle which is now widely utilized across a wide variety of disciplines, from natural sciences and medicine to engineering and computational geometry^10–18^. Preliminary analysis of the liver lobular architecture in porcine and human livers by our group suggested a relationship between the general lobular organization of liver tissue and Voronoi diagrams—further understanding of which could prove useful for the study of liver development, microanatomy, and various disease processes.

In this study, we investigate various aspects of the microanatomy of the human liver and whether the mathematical properties of Voronoi diagrams and its geometric/graph theoretical dual, Delaunay triangulation, can be used to more precisely describe classic hepatic lobules and to determine whether this mathematical tool can offer further insights to our understanding of the liver microarchitecture that could be useful in the emerging era of computational histopathology.

## Results

### Porcine livers

Two whole-slide sections of pig liver were analyzed, measuring 0.84 and 0.64 cm^2^ in total area, containing 127 and 76 lobules, and 190 and 130 portal tracts, respectively. In this species, since the boundaries of the classic hepatic lobules are demarcated by interlobular connective tissue, we were able to precisely assess the accuracy with which Voronoi diagrams describe the two-dimensional hepatic lobular organization. The classic lobular architecture of the liver largely overlapped with Voronoi diagrams obtained through any of our five methods, which showed a surface overlap area ranging from 86.7% (object edge method) to 88.6% (trial and error method) of all 203 porcine lobules by digital analysis (P = 0.007, but no statistically significant difference among trial and error, modified edge, and centroid methods).

Voronoi diagrams also fairly accurately described the overall two-dimensional/cross sectional shape, number of sides, angles, and general orientation of most lobules (Supplemental figure S2). Utilizing the trial and error method, the most common Voronoi region shape in pigs was the pentagon (representing 40% [81/203] of lobules), followed by the hexagon (32.5% [66/203] of lobules) (Supplemental figure S3). The number of sides of polygons ranged from 3 (triangles) to 8 (octagons). The average area of pig lobules in our samples was 0.69 mm^2^ (standard deviation [SD], 0.20 mm^2^) and the average circumcircle polygon diameter was 1.2 mm.

The edges of Voronoi regions either coincided or were located in close proximity with the majority of interlobular fibrous septa of cross-sections of classic lobules. In a subgroup of larger lobules, however, corresponding to “compound hepatic lobules” described by Ekataksin and Wake^19^, Voronoi diagrams often subdivided individual compound lobules (which almost invariably contained 2 or more central vein profiles) in different regions. Therefore, one or more of the Voronoi edges did not correspond to interlobular connective tissue septa in these instances (which represented 14.3% [29/203] of porcine lobules). Occasionally, very small lobule cross sections were also present—presumably representing either tangential sectioning or the terminal aspect of a lobule—and their corresponding Voronoi regions were significantly larger than the lobule profile (representing 6.9% [14/203] of porcine lobules). Examples of these structures are illustrated in Fig. 7.

### Human livers

Five whole-slide sections of human liver were analyzed, containing a total of 11.25 cm^2^ of tissue (median: 2.42 cm^2^; range, 1.46–2.88 cm^2^) and a total of 812 portal tracts (median: 170 portal tracts; range 113–217).

In humans, since the boundaries of the classic hepatic lobules are not histologically demarcated, we assessed the accuracy with which Voronoi diagrams describe the two-dimensional hepatic lobular organization based on the presence of GS-positive areas near the center of the Voronoi regions (i.e., zones 3) and the inclusion of previously annotated portal tracts within the peripheral areas of Voronoi regions (i.e., zones 1), as defined in this study. In order to accomplish this, we have created an algorithm (referred to as zonal algorithm) to subdivide each Voronoi or modified Voronoi region (depending on the Voronoi method utilized) into three different zones, analogous to the Rappaport acinus model.

The algorithm method of Voronoi generation had the best performance for portal tracts (84.4% falling within zones 1) and the modified edge method had the best performance for GS-positive areas (97.2% of positive area falling within zones 3). The total accuracy scores (encompassing zones 1 and zones 3) for each method ranged from 85.2 to 89.6% for the five methods, with the modified edge method being overall the most accurate.

Utilizing the most accurate method for each sample, the most common Voronoi region shape in humans was the heptagon (representing 34% [353/1035] of lobules), closely followed by the hexagon (32% [331/1035] of lobules) (Supplemental figure S4). The number of sides of Voronoi polygons in humans, similarly to pigs, ranged from 3 (triangles) to 8 (octagons). The average area of human lobules in our samples was 0.89 mm^2^ (standard deviation [SD], 0.51 mm^2^) and the median circumcircle polygon diameter was 1.3 mm.

## Discussion

The classic liver lobule—most clearly visualized in the normal porcine liver, in which dense and well-delineated interlobular fibrous septa linking portal tracts establish an easily identifiable boundary to each classic lobule—has traditionally been characterized and illustrated as uniform hexagonal structures, with portal tracts positioned in each of the six vertices and a central vein located in its center (Fig. 1). The human liver is generally thought to be organized in a similar fashion, although the delineation of the classic lobule cannot be properly visualized on routines stains. Functionally, in both human and non-human mammalian species, and based on the microanatomy of the classic liver lobule, hepatocytes are subdivided into three distinct zones (Rappaport lobule, Fig. 1). In spite of the typical lobular representation as juxtaposed regular hexagons, histologic examination of porcine liver sections (or human liver sections stained with zone-specific immunohistochemical markers) reveals a decidedly more complex picture: liver lobules of variable sizes and shapes—commonly but not always roughly hexagonal—with a non-uniform number of portal tracts surrounding each central vein. This relatively high degree of variability also extends to the central veins themselves, which are often somewhat eccentrically located within the liver lobules, show a seemingly haphazard orientation, and frequently bifurcate or trifurcate.

Although the three-dimensional structure of the liver lobule is fairly complex and proper orientation of portal tracts and central veins is not practically feasible histologically, evaluation of sections with the aid of zone-specific immunohistochemical markers and utilization of digital tools enabled us to hypothesize a novel concept—whereby the two dimensional classic lobular architecture of the liver is neither random nor uniform; rather, it is generally organized following the mathematical/geometric principles of Voronoi diagram and Delaunay triangulation.

Some of the basic concepts related to Voronoi diagrams were investigated as early as 1644 by the French philosopher and mathematician René Descartes, as a method of describing the distribution of matter throughout the solar system and the universe (Supplemental figure S1). This method was later applied by the German mathematician Johan Dirichlet in 1850 to the study of quadratic forms. Voronoi diagrams (also known as Voronoi tessellation, Voronoi partition, or Dirichlet tessellation) were named after the Russian mathematician Georgy Voronoy, who expanded Dirichlet’s formalized concepts of diagrams in the two- and three-dimensional cases to the n-dimensional case. A Voronoi diagram is defined as the partition of a plane with *n* generating seeds (also referred to as “sites”, “points”, or “generators”) into convex polygons (known as “regions”, or “cells”), in which each polygon has exactly one seed and every specific location within a given polygon is nearest to its generating seed than to any other seed^20–22^.

Voronoi patterns are quite ubiquitous in nature—as exemplified by the crystalloid structure of some minerals, the puzzle-like pattern of a giraffe’s fur, the delicate ridges on a dragonfly’s wings, and tortoise shell plates (Supplemental figure S5). This pattern arises in many cases due to expansion (or growth in case of biological tissue) from the “seed”/originating point of the Voronoi region outwards (Supplemental figure S6). Gomez-Galvez et al.^13^ have proposed that a three-dimensional geometrical shape named “scutoid” (resembling the scutellum of a beetle) represents the optimal configuration for energy efficiency and three-dimensional packing of epithelial cells. This unique geometrical shape was predicted by Voronoi tessellation models and subsequently verified in various types of epithelium by the same group. Voronoi diagrams have also been utilized to study the density and spatial distribution of neurons^14^, to design 3D scaffolds for bone tissue bioengineering^15^, to explain tissue self-organization and cytomorphology^16^, and to model human tumor tissue growth^17^. Voronoi diagrams are also widely utilized in the field of computational pathology and digital image analysis in histology and histopathology, especially with the purpose of outlining the otherwise difficult-to-recognize cell borders on histologic sections (both routine stains and immunostains). Its graph theoretical dual—Delaunay triangulation—is also employed in various forms of spatial analysis between cells, cell types, and microanatomic structures^23,24^.

Our data demonstrated that the overall two-dimensional lobular architecture of the liver in both pigs and humans is characterized by a pattern that closely approximates that of a Voronoi diagram, with central veins representing originating sites. This knowledge seems particularly helpful since, in humans, neither the classic lobule nor the hepatic acinus is specifically demarcated histologically, and even histochemical/ immunohistochemical zonal markers are not practically useful with respect to precise lobular delineation. Although Voronoi (and Voronoi-like) diagrams can be constructed using slightly different methods in this context, all approaches utilized by our group were able to describe the known classic lobular architecture of porcine livers with an accuracy greater than 85%, as assessed by digital image analysis and, in humans, voronoi diagrams were able to place GS-positive areas in zones 3 and portal tracts in zones 1 with an overall accuracy ranging from 85% to nearly 90%. Therefore, our data strongly indicate that the typical characterization of the two-dimensional lobular architecture of the liver as juxtaposed regular hexagons is inaccurate or, at best, oversimplified. Rather, the hepatic lobular organization is best described as a “Voronoi pattern” in which polygons with 5–7 sides predominate (Fig. 8).

In pigs, our model showed pentagons to actually represent the most common shape of Voronoi polygons modeling classic lobules (40%), followed by hexagons (32.5%), with number of sides varying from 3 to 8. These observations are essentially in agreement with early meticulous descriptions by E. G. White in 1939^25^, who also noted pentagons (47%) and hexagons (37%) to be the most common shapes among 650 adult porcine lobules, with number of sides varying from 3 to 8. Our model also showed a mean lobular area of 0.69 mm^2^ in pigs. While the area of lobules was not calculated in classic studies, the works of White^25^, Johnson^26^, and Mall^5^ mention average diameters of 1.5 mm, 1.2 mm, and 1.2 mm, respectively (compared to 1.2 mm in our model).

In humans, information regarding the shape and size of human classic lobules is fairly scarce in literature. The radius of the human lobules has been recently measured at 491 µm (diameter of approximately 1 mm) based on portal tract-central vein distances by Hall et al.^27^, which is in keeping with the previously stated lobular diameters ranging from 1.0 to 1.3 mm^28–30^. Based on these numbers, using an circumcircle radius of 0.491 to 0.65 mm for a regular heptagon (considering this as a prototypical human lobule), the resulting polygon area would be 0.65–1.15 mm^2^ (or 0.89 mm^2^ using the average [0.57 mm] of these previously reported values), which is the exact average cross-sectional area obtained by our model. Using alkaline phosphatase histochemical stain, Teutsch^31^ reported human liver lobules to be polyhedral, with seven to nine facets. In our model, the most common shapes were heptagons (34%) and hexagons (32%), with number of polygon sides varying from 3 to 8. In addition, as predicted by Voronoi tessellation, the non-equidistant central veins in sections of human livers are often eccentrically placed in their respective polygons (lobules) rather than always being at or around their center, as would be the case for a honeycomb pattern formed exclusively by regular hexagons.

The three-dimensional structure of the liver is, overall, still poorly understood. Teutsch^31^, in a 3D reconstruction of a portion of human liver, described classic lobules (referred to in his work as “primary modules”) as polyhedral structures with seven to nine facets, having heights of up to 0.9 mm and volumes of 0.1 to 0.9 mm^3^. The precise visualization and 3D reconstruction of individual liver lobules (in humans as well as in mice and rats) is further complicated by the lack of a recognizable boundary at the histologic level, hence requiring histochemical or immunohistochemical methods^31,32^. Voronoi diagrams are widely used in three-dimentional spatial partitioning, generating convex polyhedra^33^. Therefore, conceptually, Voronoi diagrams (or approximate Voronoi diagrams) could be used to attempt to mathematically model the complex 3D architecture of liver lobules. This, however, was beyond the scope of this study.

Aside from representing a more accurate descriptive model of the classic lobular architecture of the liver, the geometric properties proposed in this study also have implications to other liver unit models. Regarding liver zonation, for instance, the precise boundaries between the different zones of the Rappaport lobule can be established mathematically (or computationally) based on the location of central veins—especially if aided by central zone-specific immunostain or immunofluorescence markers. Currently, zonation of liver tissue cannot reliably be established by any method, especially in areas away from the immediate vicinity of central veins and portal tracts. As part of this study, and based on Voronoi diagrams, we have designed a digital image analysis algorithm that was able to delineate the borders of classic lobules in both pigs and humans as well as subdivide each lobule into different zones, in a fashion analogous to the Rappaport acinar model. We were also able to test the performance of our algorithm in human livers given the known location of zones 3 (GS-positive centrilobular areas) and positions of portal tracts (within zone 1). Using these structures as zonal landmarks, our best model had an accuracy of nearly 90%. Hence, this method—or future refinements thereof—could be used to more accurately and objectively study not only the size and shape of liver lobules in normal conditions (and how these may change in different diseases) but also the zonality of normal phenomena in hepatic physiology and zonal involvement by common pathologic processes such as steatosis, inflammation, necrosis, and liver fibrosis. In addition, using a computational geometry algorithmic approach, the delineation and definition of lobular zones (i.e., size and configuration of each zone) can be customized to specific needs, according to which physiologic or pathologic process is being studied.

Finally, an important implication derived from the Voronoi organization of the classic liver lobules relates to its embryology. Although the specific dynamics of hepatocyte tissue growth during embryological and post-natal development is highly complex—and beyond the scope of this study—the Voronoi organization of the classic liver lobules would indicate that cellular growth of primordial hepatocytes starts at the vicinity of the central vein and proceeds outwards radially, with portal tracts (and fibrous septa in some species) forming along the expanding edge of the nascent lobules—and eventually settling along the border of two or three classic lobular units.

In summary, our work describes the relationship between Voronoi diagrams and the liver microarchitecture in both pigs and humans. We have presented histologic and mathematical evidence that Voronoi diagrams accurately describe the basic two-dimensional organization of the normal liver. This method seems especially relevant to the study human livers, since reliable histologic landmarks of lobular boundaries are absent. We have also designed an algorithm based on Voronoi diagrams that enabled us to delineate boundaries of zones 1–3 within the classic lobules in humans, hence representing a method that would allow for more precise quantitative analysis of both physiologic and pathologic zonal processes, regardless of how, exactly, the hepatic zones are defined. Therefore, in addition to a better understanding of the liver microstructure itself, the utilization of this mathematical/computational tool opens numerous possibilities of relevant applications in the study of the normal liver and liver diseases, especially in view of the increasing utilization of digital pathology and artificial intelligence-assisted histologic evaluation.

## Materials and methods

### Histologic samples

Normal human liver samples (n = 5; 2 males and 3 females; median age 57, range 29–77) were obtained from Mayo Clinic archival surgical pathology material, utilizing sections of grossly normal liver tissue (based on gross examination of surgical specimens). Specimens were obtained from large (lobectomy) specimens performed for excision of benign (focal nodular hyperplasia, n = 2) or malignant (metastatic breast cancer, n = 1; hepatocellular carcinoma, n = 2) lesions. All patients had a single lesion within the resected specimen and at least 5 cm of surrounding normal liver, from which sections were taken. All five patients had normal liver enzymes, except one patient who had mildly elevated serum levels of aspartate aminotransferase (59 U/L) and one who had mildly elevated serum alkaline phosphatase (171 U/L). None of the patients had clinical history of liver diseases, clinical signs of portal hypertension, or received pre-surgical chemo-radiation therapy for the hepatic lesion. Normal adult Landrace porcine liver sections were obtained from archival histology material from the Mayo Clinic William J. von Liebig Center for Transplantation and Clinical Regeneration.

H&E, trichrome, and reticulin stains were performed on all samples with standard protocols, reviewed by a liver pathology specialist, and showed no fibrosis, nodularity, or other histopathologic abnormalities, aside from very mild (less than 5%) steatosis in two of the human samples. Immunohistochemical studies for the centrilobular marker glutamine synthetase (GS) (monoclonal antibody, clone GS-6; Millipore, Temecula, CA, USA) were performed on all samples.

Human and tissue samples were obtained in accordance to the regulations and with the approval of Mayo Clinic Institutional Review Board. All human tissue samples we have utilized for this study are from archived material (i.e. left over tissue previously obtained for medical procedures and already thoroughly tested/evaluated from a clinical perspective). None of the samples were obtained exclusively or specifically for the purposes of this study. Consent waiver for this study was approved by Mayo Clinic Institutional Review Board. Animal samples were obtained in accordance to the regulations and with the approval of the American Association for Laboratory Animal Science (IACUC).

### Digital analysis

Glass slides were scanned and imaged using the Aperio (Leica) ScanScope AT Turbo Instrument. Each slide was scanned at × 40 magnification on the Aperio ScanScope AT Turbo brightfield instrument (Leica Biosystems) at a resolution of 0.50 μm per pixel. ImageScope and eSlide Manager (Leica Biosystems) were utilized to view the digital images. Whole slide images (WSI) and still images were used for histologic annotations and digital image analysis using QuPath v-0.2.0-m12 and Fiji ImageJ 1.52p. Voronoi diagram composite images were analyzed using OpenCV version 4.3 and Shapely vesion 1.7, both in Python 3 version 3.6.

### Generation of Voronoi diagrams

Voronoi diagrams and, when applicable, Delaunay triangulation, were digitally generated using GS-positive perivenular areas as references for the position of generating points using the “Delaunay Voronoi” plugin in Fiji ImageJ 1.52p. This software permits the placement of points at any location within a given image (and subsequent adjustment of their position if needed) and interactive generation of Voronoi diagrams and/or Delaunay triangulation based on the position of these points. Voronoi diagrams were generated using five different methods.

**Figure 2:**
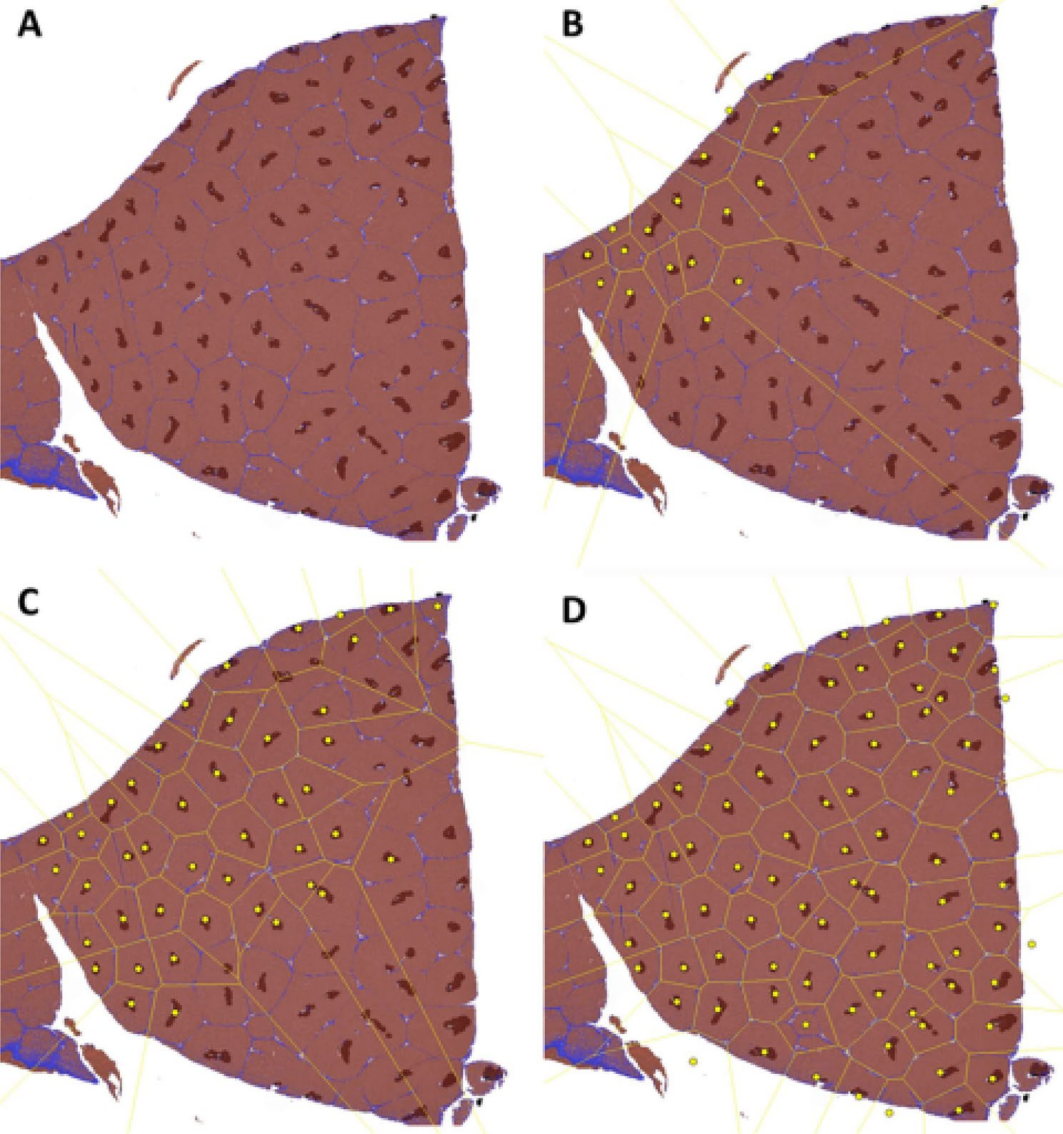
“Trial and error” method in a section of porcine liver (AI-assisted colorized Masson trichrome/glutamine synthetase immunostains composite image). Utilizing an interactive Voronoi digital tool (Fiji ImageJ Voronoi Delaunay plugin), Voronoi sites (yellow dots) were placed at the location of central veins. As Voronoi regions were generated, the location of sites was adjusted, as needed, for maximum overlap of Voronoi edges with the boundary of lobules (interlobular fibrous tissue). Original image (**A**). Initiation of the interactive Voronoi process (**B**). Continuation of interactive Voronoi process (**C**). Final Voronoi diagram (**D**). Sofware utilized: QuPath v-.2.0-m12 (Bankhead, P. et al. (2017). QuPath: Open source software for digital pathology image analysis. Scientific Reports) and Fiji ImageJ 1.52p (Schindelin, J.; Arganda-Carreras, I. & Frise, E. et al. (2012), "Fiji: an open-source platform for biological-image analysis", Nature methods 9(7): 676–682).

**Figure 3:**
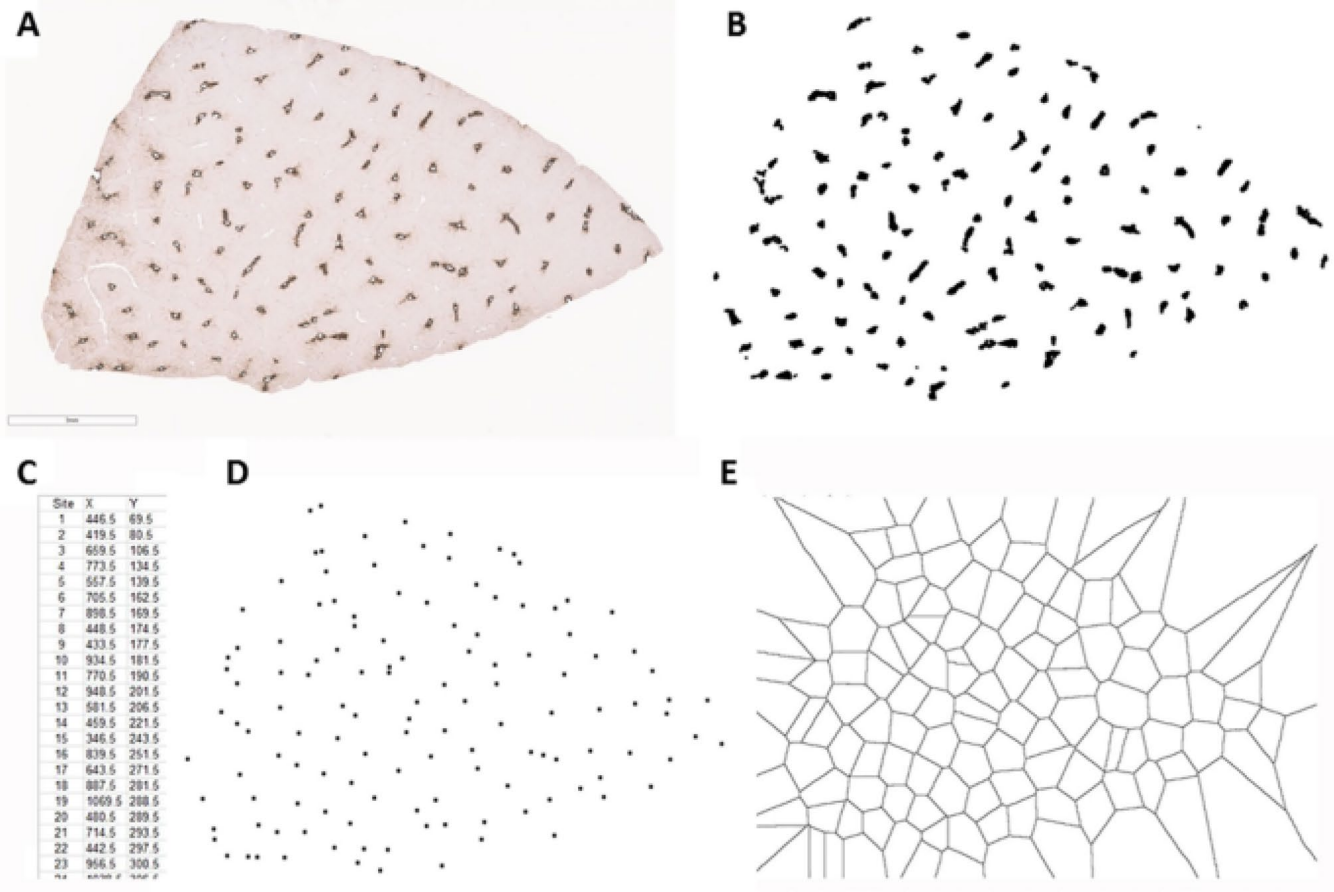
“Centroid” approach for obtaining a Voronoi diagram based on the location of central veins in a section of normal porcine liver. The process starts with the centrilobular/zone 3 marker glutamine synthetase (GS) immunostain (**A**). GS-positive areas are segmented and transformed into a binary image (**B**). Centroid coordinates are obtained (**C**). Location of centroids are plotted (**D**). Voronoi diagram is generated (**E**) for subsequent overlay with trichrome-GS composite image (not shown). Software utilized: Fiji ImageJ 1.52p (Schindelin, J.; Arganda-Carreras, I. & Frise, E. et al. (2012), "Fiji: an open-source platform for biological-image analysis", Nature methods 9(7): 676–682).

**Figure 4:**
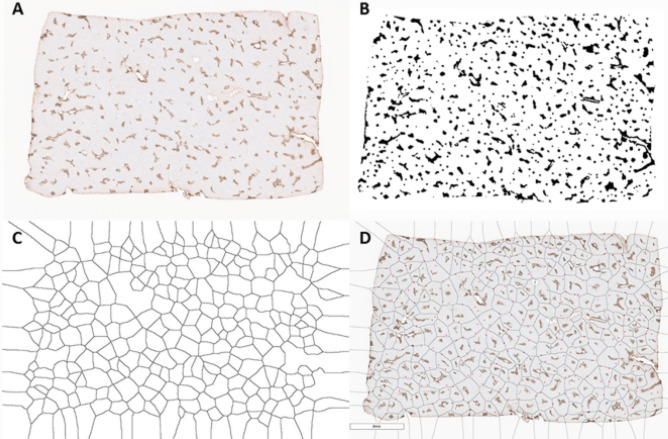
“Object edge” approach for obtaining a Voronoi diagram based on the location of central veins on glutamine synthetase (GS) immunostains in a section of normal human liver. Original GS immunostain (**A**) and binarization of GS-positive zone 3 areas (**B**). Voronoi-like diagram is generated based on the edges of each object (rather than a single point) (**C**). Diagram is combined with GS immunostains whole slide scan image; portal tracts have been previously annotated as red stars on the original immunostains image; notice that the large majority of portal tracts are located along the edges of Voronoi regions (**D**). Software utilized: Fiji ImageJ 1.52p (Schindelin, J.; Arganda-Carreras, I. & Frise, E. et al. (2012), "Fiji: an open-source platform for biological-image analysis", Nature methods 9(7): 676–682).

**Figure 5:**
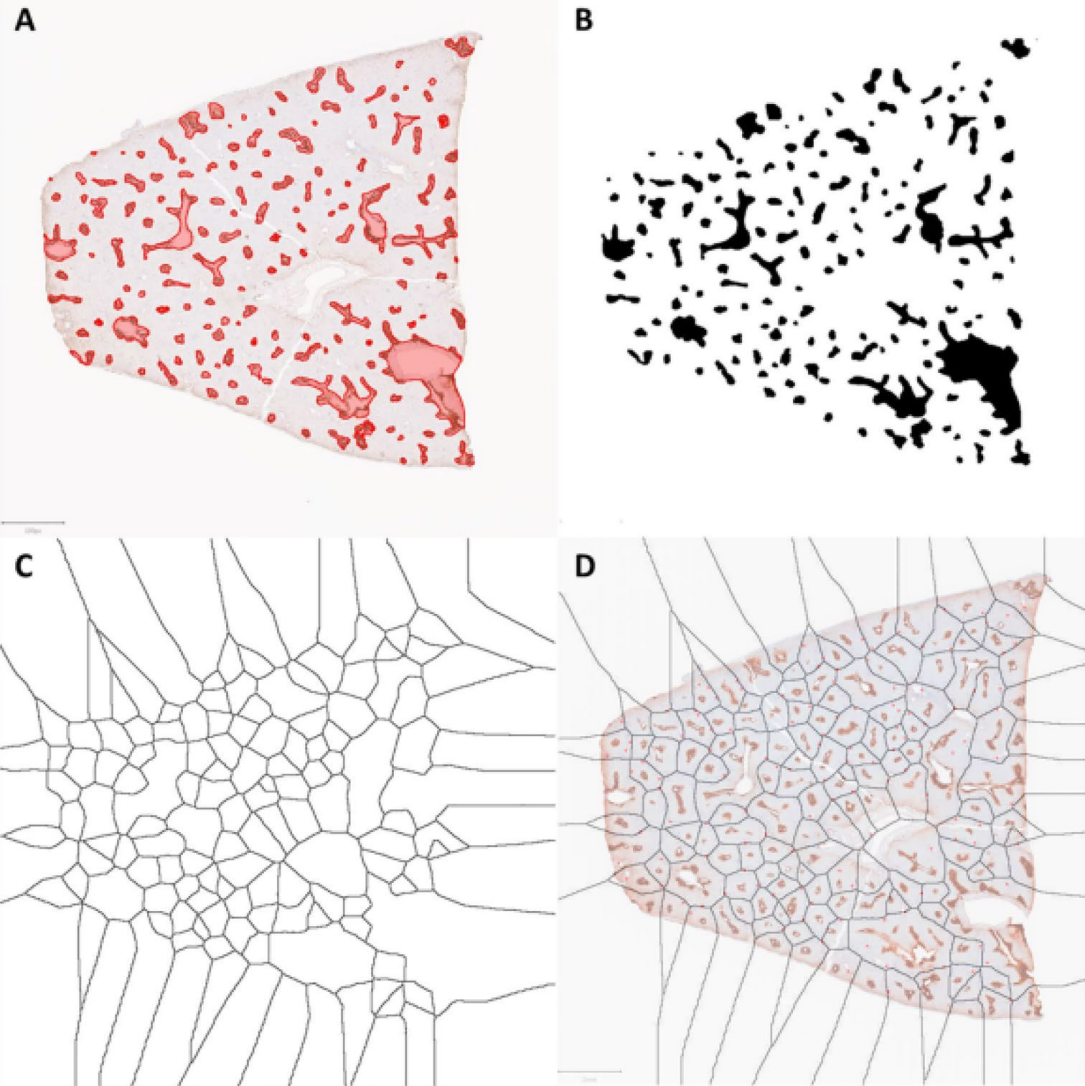
Modified object edge method: digital annotation of glutamine synthetase (GS) immunostains of normal human liver (**A**), binary transformation (**B**), generation of Voronoi-like diagram based on object edges (**C**) and overlay with original GS immunostain with annotated portal tracts (**D**). Software utilized: QuPath v-.2.0-m12 (Bankhead, P. et al. (2017). QuPath: Open-source software for digital pathology image analysis. Scientific Reports) and Fiji ImageJ 1.52p (Schindelin, J.; Arganda-Carreras, I. & Frise, E. et al. (2012), "Fiji: an open-source platform for biological-image analysis", Nature methods 9(7): 676–682).

1. “Trial and error” approach: Using Fiji ImageJ Delaunay Voronoi plugin and whole-section images obtained from WSI files, dots were placed over recognizable central veins or GS-positive areas in both pig and human liver sections. Trichrome-GS composite images were used for pig livers and annotated GS images were used in human livers. If no recognizable central vein was present within a given porcine lobule, a point was placed in its center. The position of points were then adjusted as needed, on a trial and error basis, so that the edges of the Voronoi diagram overlapped maximally with the interlobular fibrous tissue of liver lobules (in porcine livers) and with surrounding previously annotated portal tracts (in human livers) (Fig. 2).
2. “Centroid”approach: Whole-section still images from WSI scans of GS immunohistochemistry slides were utilized and further processed digitally using Fiji ImageJ (8-bit conversion, followed by image thresholding – Image > Adjust > Threshold [using “default” and “red” settings]). The image was then converted to binary (Process > binary > Make binary) and background noise was cleared (Process > Noise > Despeckle). Centroid coordinates were then obtained (Analyze > analyze particle, with “centroid” checked under “Set Measurements”). Voronoi diagrams were then obtained utilizing centroid locations as points (Fig. 3).
3. “Object edge” approach: Images were processed in a manner identical to that described for the centroid approach (except for the centroid, coordinates, and plotting steps). The resulting image was then converted to a Voronoi-like pattern (Process > Binary > Voronoi), whereby lines with equal distance to the borders (rather than centroid) of the two nearest particles are generated. Thus, the resulting Voronoi-like partition, similarly to a true voronoi diagram, includes all points that are nearer to the edge of its generating particle than to the edge of any other particle. However, given the fact that most of the particles were not single points (but, rather, irregular regions of varying sizes), the resulting partition does not follow all the defining features of a Voronoi diagram from a mathematical standpoint, therefore being referred to here as a Voronoi-like diagram (Fig. 4).
4. “Modified object edge” approach: Whole-section still images from WSI scans of GS immunohistochemistry slides were utilized. GS-positive perivenular areas and central veins were manually annotated as objects using QuPath v-0.2.0-m12, then further processed using “residual” under Brightness and Contrast” for background exclusion. The resulting image was exported to ImageJ for segmentation (8-bit conversion, then Image > Threshold) and generation of Voronoi-like diagram (Process > Binary > Voronoi) (Fig. 5).
5. Algorithm approach: The algorithm utilized the Python3 language as well as OpenCV, an image-processing library for Python3 and Scipy.spatial library for Voronoi diagram generation. Still images from WSI scans of trichrome and GS immunohistochemistry slides (annotated GS stains for humans and trichrome and trichome-GS composite images for pigs) were utilized. Central veins were annotated and transformed into objects using OpenCV. The GS-positive perivenular areas were recognized by OpenCV as objects and transformed into single points (object centroids). First, a Voronoi diagram based on the location of centroids of GS-positive perivenular areas was generated. The diagram was then refined by adjusting the position of both the polygon edges and vertices in order for them to maximally match the position of interlobular fibrous septa (in pig livers) and annotated portal tracts (in humans). In order to preserve a structure at least closely approximating a Voronoi diagram, a depth-first search error metric that measures the mismatch between the original Voronoi diagram and the adjusted diagram was obtained, with the a maximum allowed non-overlapping area of 5%. In addition to the depth-first search, a clustering algorithm was also used to separate large regions (suggesting the presence of more than one GS-positive perivenular area within the region) into smaller regions (including new Voronoi sites). The resulting image of a Voronoi-like diagram was generated using Matplotlib, a Python3 library for generating images.

**Figure 6:**
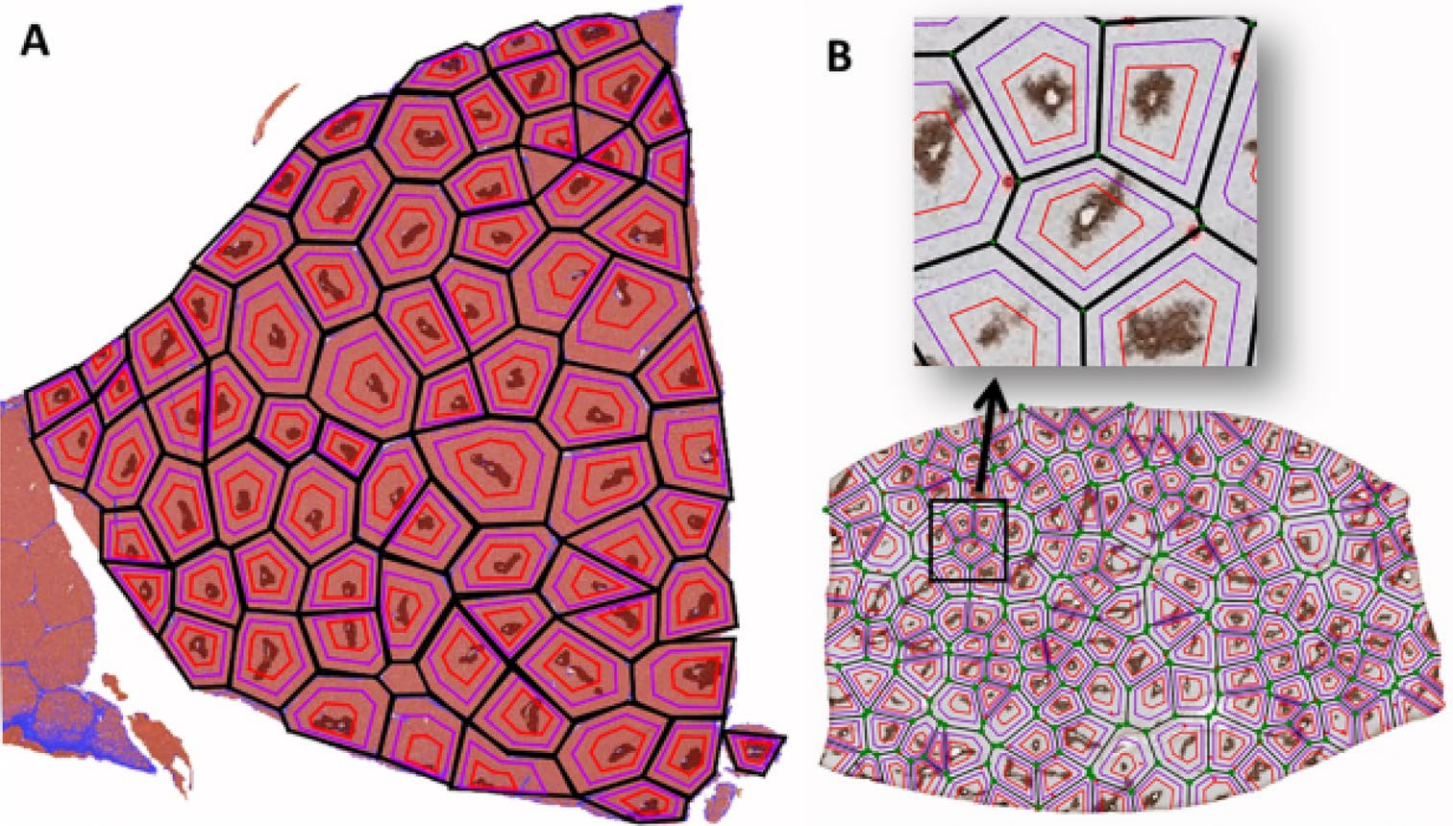
Voronoi diagram through the algorithm approach on a pig liver section (**A**) (black lines) and human liver section (**B**). Zonation algorithm further divides each lobule into three zones. Purple lines represent the border between zones 1 and 2, while red lines mark the border between zones 2 and 3 (**A**,**B**). Software utilized: QuPath v-.2.0-m12 (Bankhead, P. et al. (2017). QuPath: Open-source software for digital pathology image analysis. Scientific Reports) and Fiji ImageJ 1.52p (Schindelin, J.; Arganda-Carreras, I. & Frise, E. et al. (2012), "Fiji: an open-source platform for biological-image analysis", Nature methods 9(7): 676–682). Voronoi algorithm created in OpenCV version 4.3 and Shapely vesion 1.7, both in Python 3 version 3.6.

We have also created an algorithm to subdivide each Voronoi or modified Voronoi region into three concentric zones, analogous to the Rappaport acinus model. The zonal algorithm utilized the Python3 language and OpenCV. The general location of the GS-positive perivenular areas were recognized using OpenCV. Within each Voronoi region pertaining to a particular GS-positive area, zone 3 was defined as the region with maximum coverage of GS-positive areas and minimal coverage of GS-negative areas that maintains the same polygon configuration as its corresponding Voronoi region. Zone 1 was defined as the area between the edges of each Voronoi region and a line that runs at the midpoint between the Voronoi edge and the border of zone 3. The intermediate area between zone 3 and zone 1 (of equal width to zone 1) represented zone 2. The resulting image of the algorithm's output was generated using Matplotlib, a Python3 library for generating images. An example of Voronoi diagram by the algorithm approach and zonation is shown in Fig. 6.

### Statistical analysis

**Figure 7:**
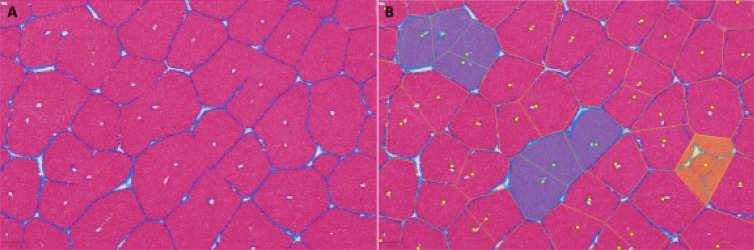
Artificial intelligence-assisted recognition of hepatocytes and fibrous tissue with digital colorization, for better visualization of lobular architecture at 20 × magnification, of a section of porcine liver (**A**). The same image with superimposed Voronoi diagram, showing compound lobules (examples highlighted in semi-translucent blue) with multiple central vein profiles (**B**). Small lobular cross sections (likely representing the tip of a lobule or a tangential section) highlighted in semi-translucent yellow (**B**). Software utilized: QuPath v-.2.0-m12 (Bankhead, P. et al. (2017). QuPath: Open source software for digital pathology image analysis. Scientific Reports) and Fiji ImageJ 1.52p (Schindelin, J.; Arganda-Carreras, I. & Frise, E. et al. (2012), "Fiji: an open-source platform for biological-image analysis", Nature methods 9(7): 676–682).

**Figure 8:**
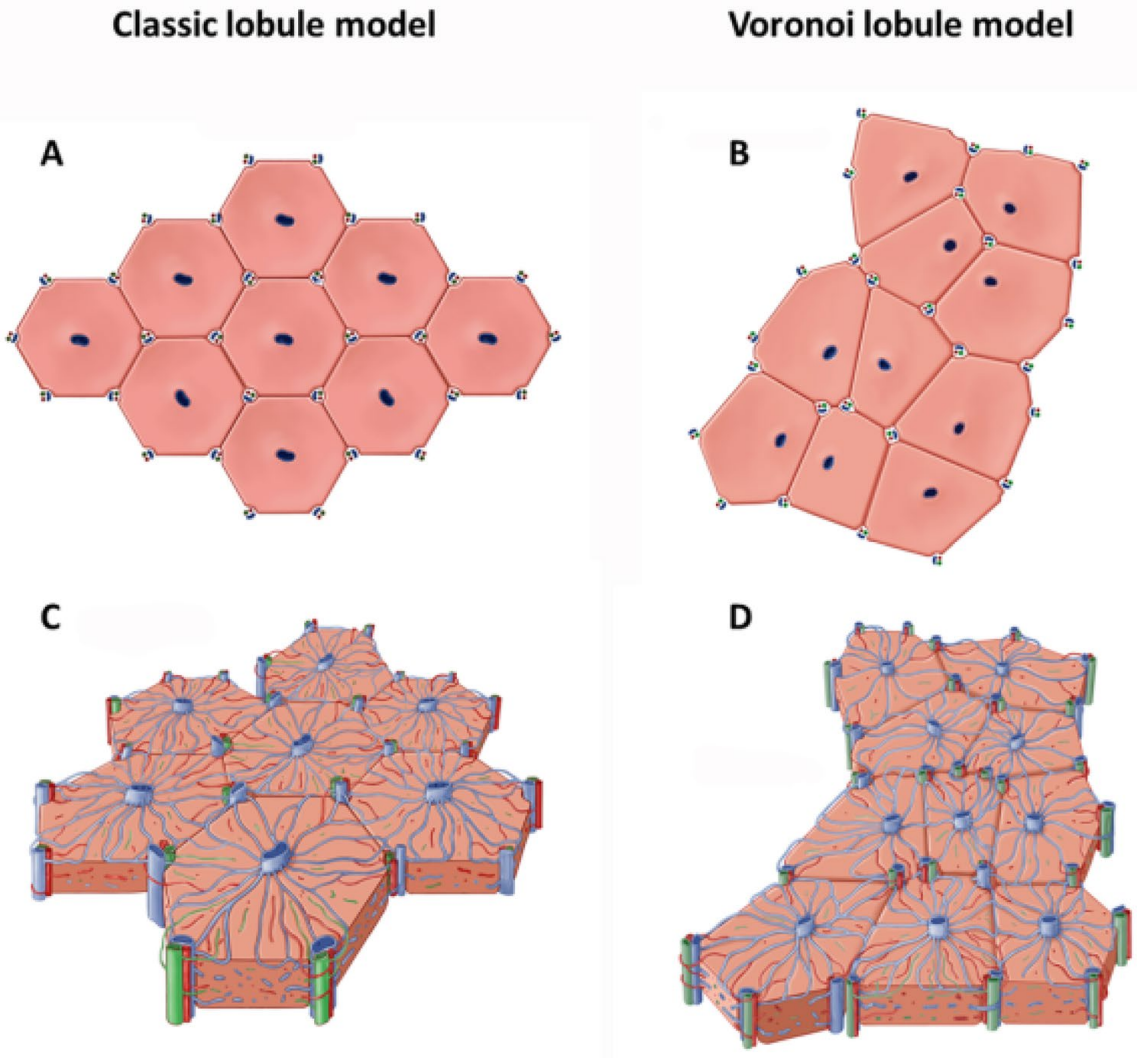
Comparison between the typical representation of the classic lobule model (**A**,**C**) formed by juxtaposed regular hexagons and the Voronoi model (**B**,**D**) characterized by a Voronoi diagram pattern. Created by Mayo Clinic Medical Illustration.

Descriptive statistics were presented using average and standard deviation or median and interquartile range for continuous data with normal and non-normal distribution, respectively. Percentages were used for categorical data. Groups were compared using one-way analysis of variance (ANOVA) and t-test. Normal distribution was tested using the Shapiro–Wilk test (MedCalc Software, Ostend, Belgium). A *P*-value < 0.05 was considered statistically significant (Figs. 7, 8).

## Supplementary Information

Supplementary Information
