# Supplementary material for: The Voronoi theory of the normal liver lobular architecture and its applicability in hepatic zonation
Source: Sci Rep. 2021 Apr 29;11:9343. doi: 10.1038/s41598-021-88699-2 (PMC8085188; doi:10.1038/s41598-021-88699-2)
Supplement: Supplementary file 1 — Supplementary Information [file 41598_2021_88699_MOESM1_ESM.pdf]

The Voronoi theory of the normal liver lobular architecture and its applicability in hepatic zonation

Lau C[1], Kalantari B[1], Batts KP[2], Ferrell LD[3], Nyberg SL[4], Graham RP[5], Moreira RK[5*].

1. Department of Computer Science, Rutgers University, Brunswick, NJ, USA
2. Allina Health, Minneapolis, MN, USA
3. Department of Pathology, University of California, San Francisco, CA, USA
4. Department of Surgery, Division of Transplantation Surgery, Mayo Clinic, Rochester, MN, USA
5. Department of pathology and Laboratory medicine, Mayo Clinic, Rochester, MN, USA*

*Corresponding author: Roger K. Moreira, M.D. Email: Moreira.roger@mayo.edu

Supplemental figures and captions:

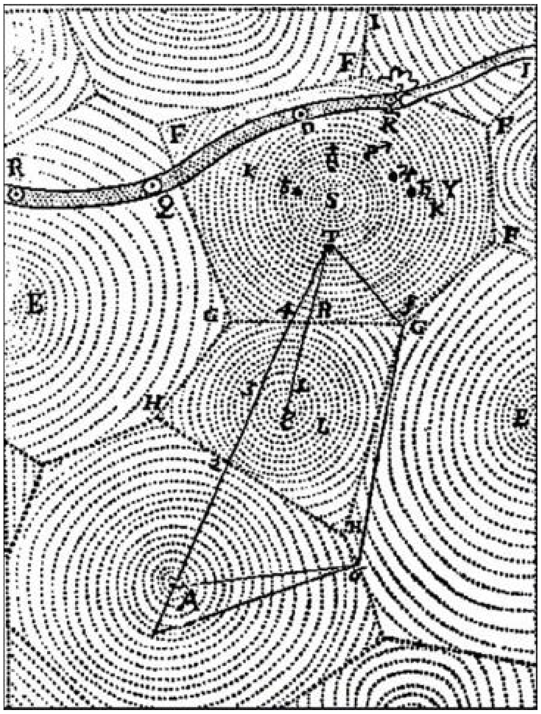

Supplemental figure 1: Illustration by the French philosopher and mathematician René Decartes in *Principia Philosophia*, 1644 – the decomposition of the universe into vortex systems, representing the first historical consideration of Voronoi diagrams.

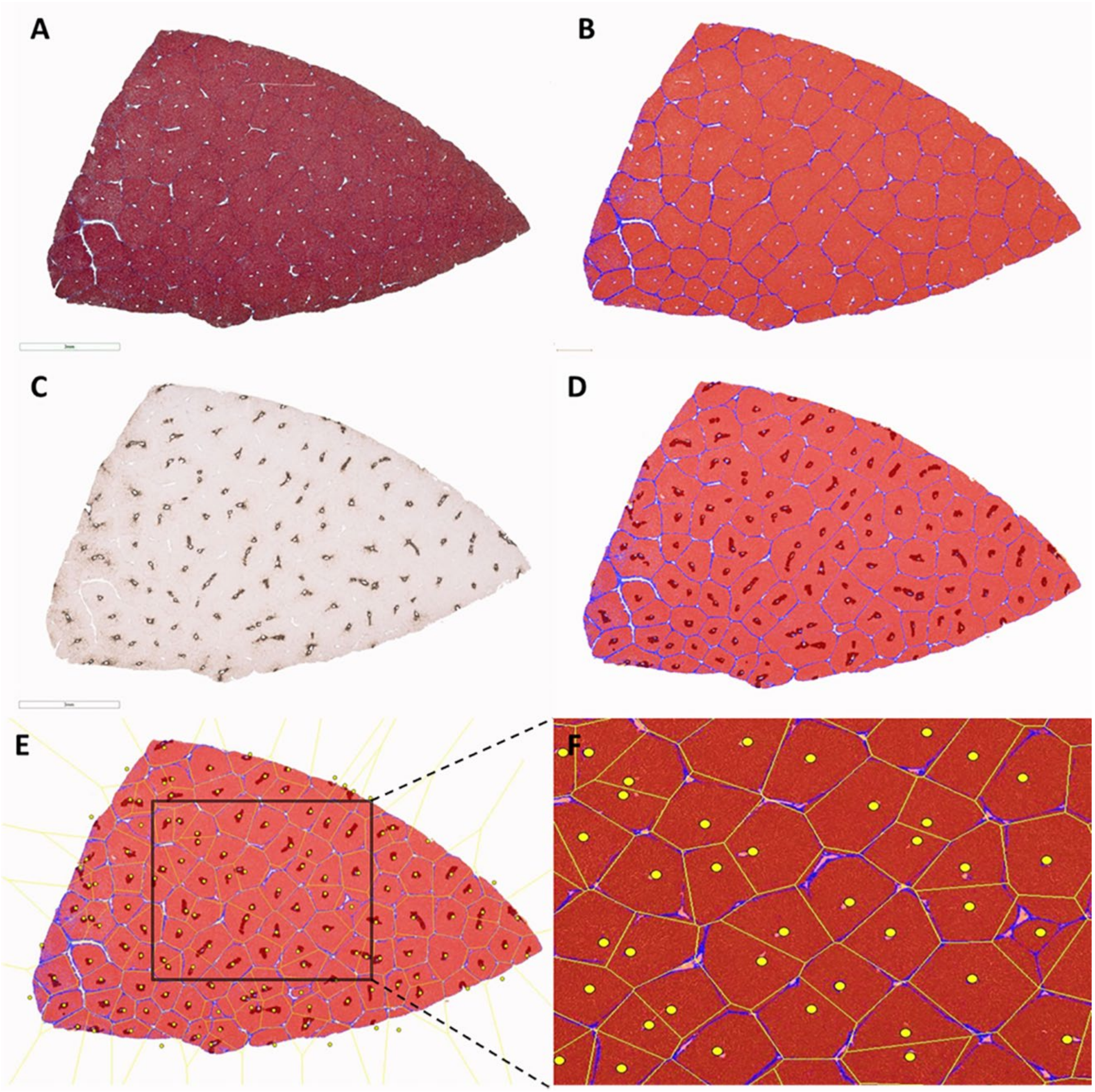

Supplemental figure 2. Whole slide image of a section of porcine liver (A-E). Original Masson trichorme stain (A). Artificial intelligence-assisted recognition of hepatocytes and fibrous tissue with digital colorization, for better visualization of lobular architecture at very low magnification (B). Glutamine synthetase immunostains (C). Colorized trichrome stain- glutamine synthetase immunostains composite image (D). Colorized trichrome stain-glutamine synthetase immunostains composite image with superimposed Voronoi diagram (yellow lines) and Voronoi sites (yellow dots) (E), with higher magnification view (F). Software utilized: QuPath v-.2.0-m12 (Bankhead, P. et al. (2017). QuPath: Open source software for digital pathology image analysis. Scientific Reports) and Fiji ImageJ 1.52p (Schindelin, J.; Arganda-Carreras, I. & Frise, E. et al. (2012), "Fiji: an open-source platform for biological-image analysis", Nature methods 9(7): 676-682).

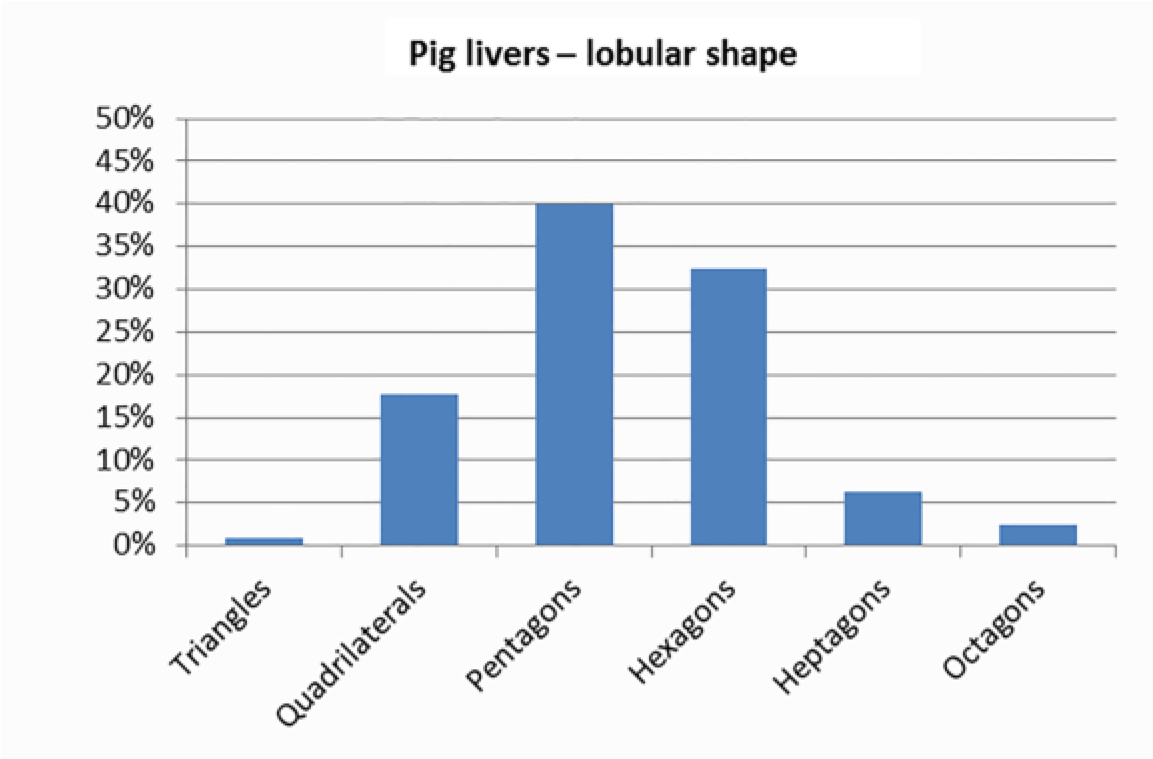

Supplemental figure 3. Frequency of polygons describing the two dimensional shape of porcine liver lobules on histologic sections.

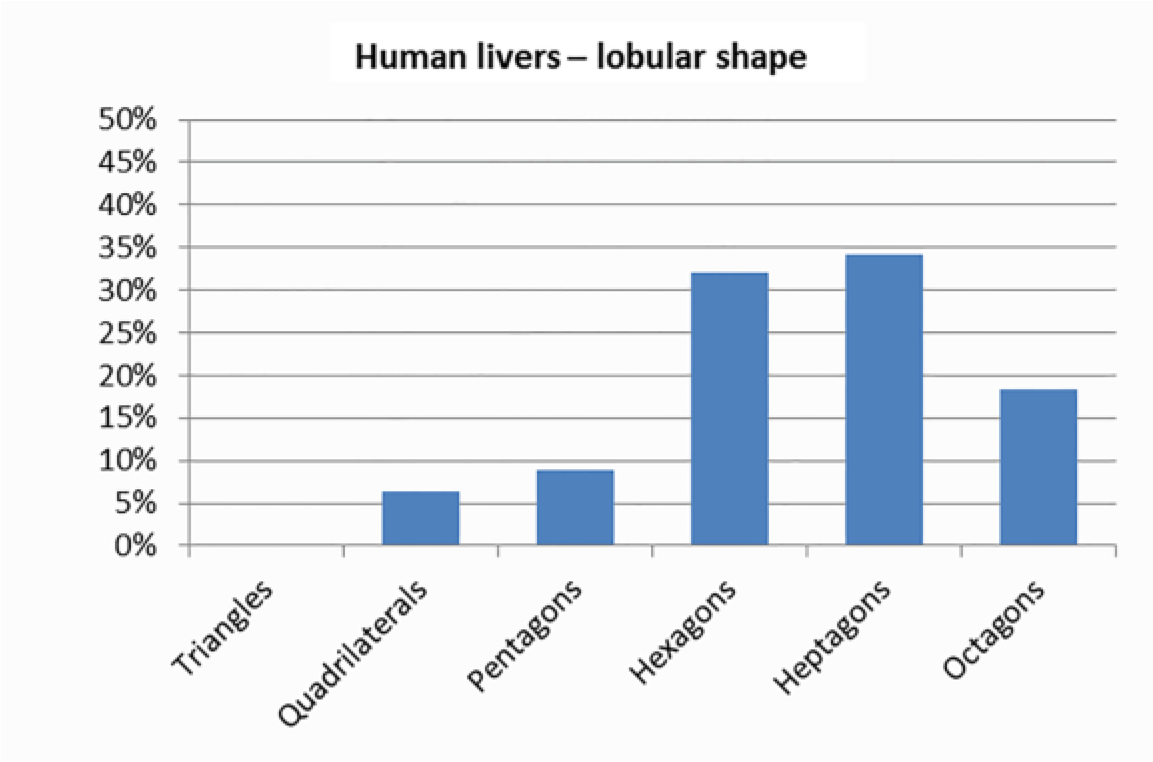

Supplemental figure 4. Frequency of polygons describing the two dimensional shape of human liver lobules on histologic sections.

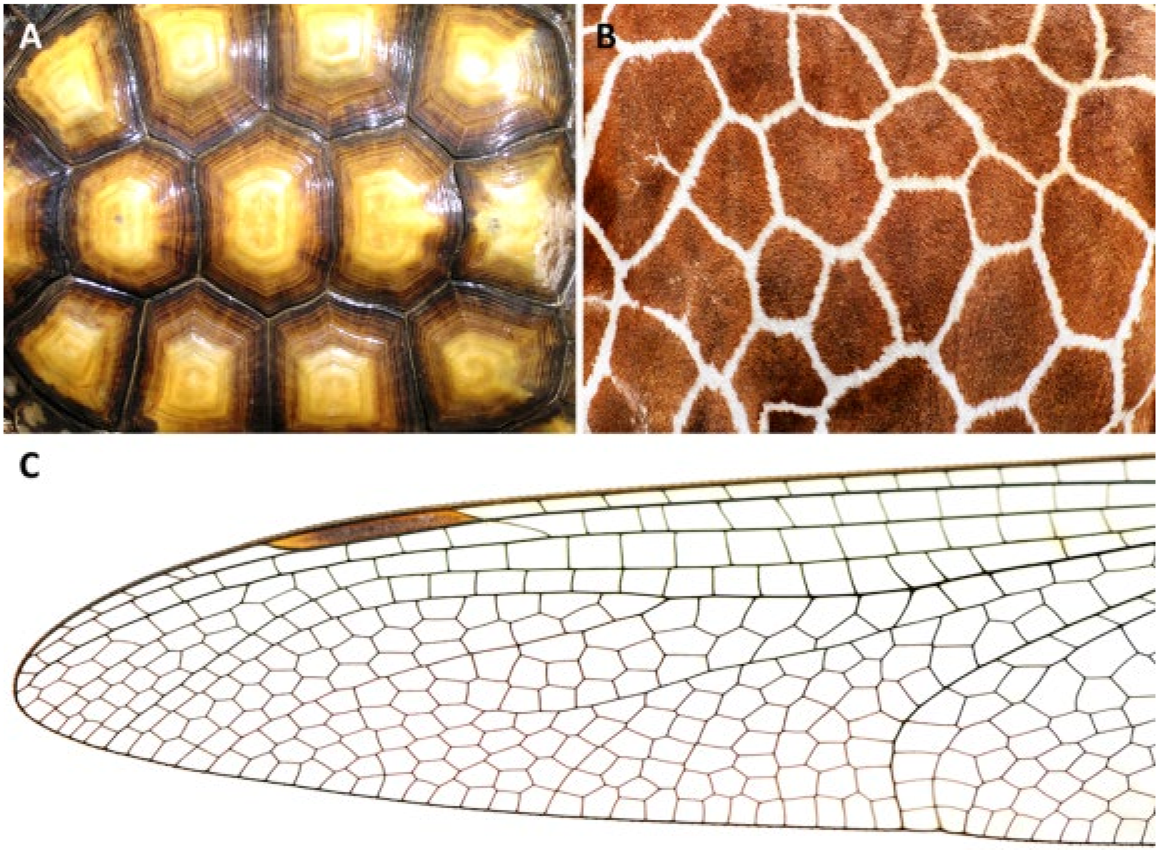

Supplemental figure 5: Examples of Voronoi patterns in nature. African spurred tortoise (*Centrochelys sulcate*) shell scutes (A), giraffe fur (B), and dragonfly wing (C). Growth of each unit takes place until their respective edges press against the expanding edges of adjacent units, in a process analogous to that illustrated in supplemental figure 6.

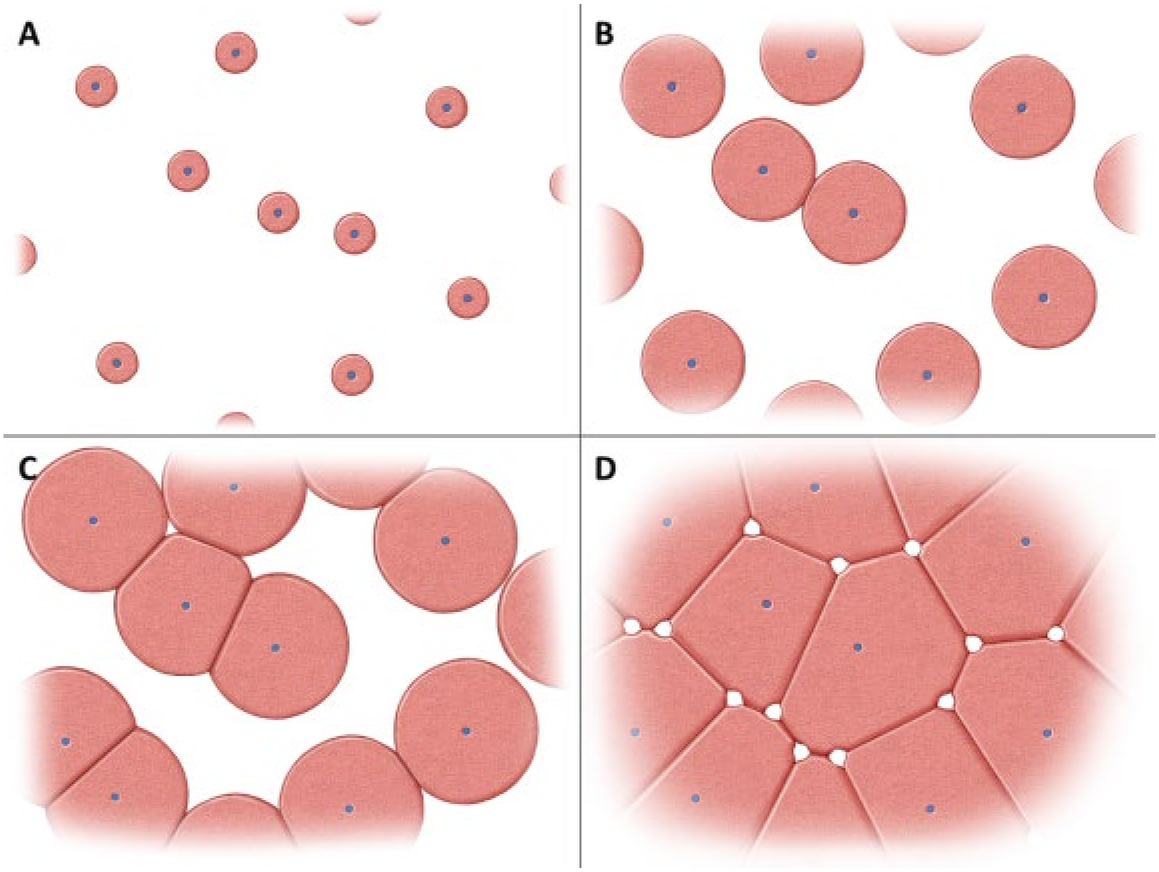

Supplemental figure 6: Formation of Voronoi regions as a result of expansion of structures until their respective borders collide and form straight lines. Created by Mayo Clinic Medical illustration.
